# Tetra­methyl 1,1,2-triphenyl-2*H*-1λ^5^-phosphole-2,3,4,5-tetra­carboxyl­ate

**DOI:** 10.1107/S1600536810037827

**Published:** 2010-10-09

**Authors:** Krzysztof K. Krawczyk, Krystyna Wojtasiewicz, Jan K. Maurin, Ewa Gronowska, Zbigniew Czarnocki

**Affiliations:** a Faculty of Chemistry, University of Warsaw, Pasteura 1, 02-093 Warsaw, Poland; b National Medicines Institute, Chełmska 30/34, 00-725 Warsaw, Poland; c Institute of Atomic Energy, 05-400 Otwock-Świerk, Poland

## Abstract

The title compound, C_30_H_27_O_8_P (1), was formed as one of two products {(1) and (2) [Krawczyk *et al.* (2010 ▶). *Acta Cryst.* E**66** (cv2753)]} in the reaction of dimethyl acetyl­enedicarboxyl­ate with triphenyl­phosphine. The mol­ecule of (1) consists of a five-membered ring, in which the P atom is incorporated. One of the phenyl groups of the triphenyl­phosphine migrated to a vicinal C atom during the reaction. The five-membered ring of (1) is corrugated [r.m.s. deviation = 0.0719 (8) Å], whereas that in compound (2) is planar, the r.m.s. deviation being only 0.009 (2) Å.

## Related literature

For general background to derivatives of dimethyl­enesuccinic anhydride (fulgides), see: Hadjoudis & Mavridis (2004 ▶); Gordaliza *et al.* (1996 ▶); Datta *et al.* (2001 ▶); Stobbe (1893 ▶); Maercker (1965 ▶); Shaw *et al.* (1967 ▶). For a detailed study of adduct formation from triaryl­phosphines and acetyl­ene­dicarboxyl­ate, see: Waite *et al.* (1971 ▶). For related structures, see: Spek (1987 ▶); Thomas & Hamor (1993 ▶); Krawczyk *et al.* (2010 ▶). 
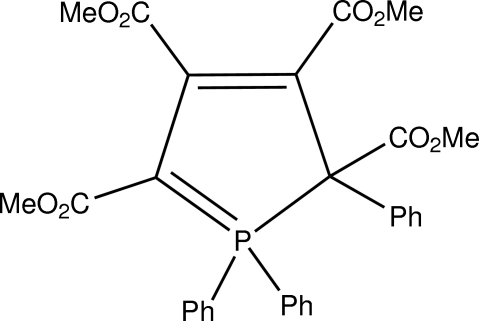


## Experimental

### 

#### Crystal data

C_30_H_27_O_8_P
*M* *_r_* = 546.49
Triclinic, 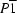

*a* = 10.445 (6) Å
*b* = 10.897 (4) Å
*c* = 13.778 (4) Å
α = 73.93 (3)°
β = 72.54 (4)°
γ = 69.24 (4)°
*V* = 1373.0 (10) Å^3^
*Z* = 2
Cu *K*α radiation
μ = 1.32 mm^−1^
*T* = 293 K
0.20 × 0.12 × 0.04 mm

#### Data collection

Oxford Diffraction Xcalibur diffractometer with Ruby CCD
Absorption correction: analytical (*CrysAlis RED*; Oxford Diffraction, 2006 ▶) *T*_min_ = 0.714, *T*_max_ = 0.885
20877 measured reflections
5207 independent reflections
4503 reflections with *I* > 2σ(*I*)
*R*_int_ = 0.032

#### Refinement

*R*[*F* ^2^ > 2σ(*F* ^2^)] = 0.041
*wR*(*F* ^2^) = 0.128
*S* = 1.10
5207 reflections
352 parameters
H-atom parameters not refined
Δρ_max_ = 0.33 e Å^−3^
Δρ_min_ = −0.24 e Å^−3^

### 

Data collection: *CrysAlis CCD* (Oxford Diffraction, 2006 ▶); cell refinement: *CrysAlis RED* (Oxford Diffraction, 2006 ▶); data reduction: *CrysAlis RED*; program(s) used to solve structure: *SHELXS97* (Sheldrick, 2008 ▶); program(s) used to refine structure: *SHELXL97* (Sheldrick, 2008 ▶); molecular graphics: *SHELXTL-NT* (Sheldrick, 2008 ▶); software used to prepare material for publication: *SHELXL97*.

## Supplementary Material

Crystal structure: contains datablocks paper, I. DOI: 10.1107/S1600536810037827/cv2752sup1.cif

Structure factors: contains datablocks I. DOI: 10.1107/S1600536810037827/cv2752Isup2.hkl

Additional supplementary materials:  crystallographic information; 3D view; checkCIF report

## Figures and Tables

**Fig. 1.**
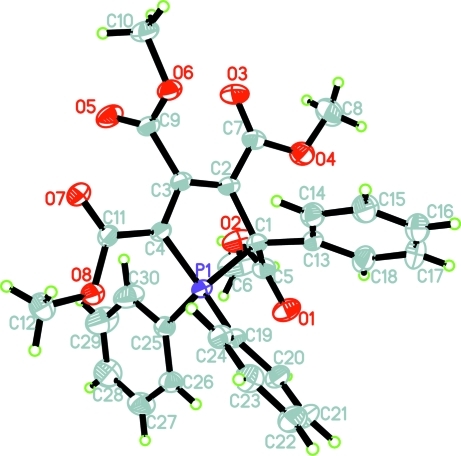
Molecular structure of (1) showing the atomic labelling and 30% probability displacement ellipsoids.

## supplementary crystallographic information

### Comment

Several derivatives of dimethylenesuccinic anhydride (fulgides) have been the subject of intensive studies due to their photochromic properties (Hadjoudis & Mavridis, 2004). As early as in 1893 Stobbe (Stobbe, 1893) discovered a very effective synthetic procedure leading to *E*,*E*-diarylfulgides. The double Stobbe condensation, after some minor modifications (Gordaliza *et al.*, 1996) still remains the method of choice in the construction of fulgide-type compounds (Datta *et al.*, 2001). However, considering some disadvantages of this procedure, *e.g.* a need for the use of strong bases, which may cause resinification of some aldehydes, there have been continuous efforts towards development of alternative approaches. Especially the Wittig reaction (Maercker, 1965) between dialkyl bis[triphenylphosphoranylidene]succinates and the appropriate benzaldehydes appeared to be of particular value. The ylide component of the Wittig reaction seemed to be easily accessible by the condensation between triphenylphosphine and dialkyl acetylenedicarboxylate. However, this reaction, performed in diethyl ether, gave tetramethyl 1,1,2-triphenyl-2*H*-1λ^5^-phosphole-2,3,4,5-tetracarboxylate (1) and another adduct - trimethyl-3-methoxy-4-oxo-5-triphenylphosphoranylidenecyclopent-1-ene- 1,2,3-tricarboxylate in 42% and 21% yield respectively (Shaw *et al.*, 1967). We found, that when dry toluene was used as a solvent, and the reaction was performed at -78°C, (1) was formed in 63% yield, and the other adduct in 28% yield. In the present comunication we report on the crystal structure of compound (1). This structure was already proposed in 1971 (Waite *et al.*, 1971) on the basis of spectral data. The crystal structure of the other adduct could also be determined *via* single-crystal diffraction (Krawczyk *et al.*, 2010).

In compound (1) (Fig. 1)  two acetyl groups at C2 and C4, respectively, are almost co-planar with the five-membered ring with the dihedral angle of 11.6 (1) and 8.47 (9)°, respectively, whereas the two remaining acetyl groups at C1 and C3 are strongly rotated from the ring plane (the dihedral angles of 67.(1) and 80.51 (8)°, respectively). The phenyl rings bonded to the phosphorous atoms in (1) have similar conformations to that observed at room temperature for the parent triphenylphosphine in both polymorphic structures (Spek, 1987; Thomas & Hamor, 1993) assuring the lowest repulsion of the neighboring fragments.

### Experimental

A mixture of acetylenedicarboxylate (0.5 g, 3,52 mmol) in 3 ml of dry toluene was placed in a two-neck round bottom flask, and cooled to -78°C (solid CO_2_/acetone bath) with stirring. The solution of triphenylphosphine (0.47 g, 1.80 mmol) in 3 ml of dry toluene was then added dropwise under argon during 20 min. The reaction was then left to reach slowly room temperature overnight. After evaporation of the solvent under reduced pressure, the remaining oil was dissolved in ethyl acetate and purified by column chromatography (Merck silica gel, 230 - 400 mesh, ethyl acetate, and then ethyl acetate/methanol 19:1 as eluent) to obtain tetramethyl-1,1,2- triphenyl-2*H*-1λ^5^-phosphole-2,3,4,5-tetracarboxylate (1) and trimethyl-3-methoxy-4-oxo-5-triphenylphosphoranylidenecyclopent-1- ene-1,2,3-tricarboxylate (2). Both products could be easily recrystallized from ethyl acetate/diethyl ether. The 2*H*-phosphole (1) (0.61 g, 63%) had Rf = 0.46 (ethyl acetate) and a melting point of 253–255°C (Waite, *et al.*1971). The second eluted product - (2) (0.27 g, 28%) - showed a green fluorescence in UV light (λ = 365 nm), had Rf = 0.18 (ethyl acetate) and melted at 243–244°C [(Waite *et al.*, 1971), m.p. 222–224°C]. The single-crystal of (1) was obtained by slow evaporation of its ethyl acetate/hexane solution.

### Refinement

H atoms were placed in calcluated positions and were included in the refinement with *U*_iso_(H) = 1.2*U*_eq_(C) [1.5 in the case of methyl groups H atoms]. Isotropic displacement parameters for hydrogen atoms bonded to either oxygen or nitrogen atoms were refined independently.

### Crystal data

**Table:**

C_30_H_27_O_8_P	*F*(000) = 572
*M**_r_* = 546.49	*D*_x_ = 1.322 Mg m^−^^3^
Triclinic, *P*1	Melting point: 527 K
*a* = 10.445 (6) Å	Cu *K*α radiation, λ = 1.54184 Å
*b* = 10.897 (4) Å	Cell parameters from 13436 reflections
*c* = 13.778 (4) Å	θ = 3.4–70.3°
α = 73.93 (3)°	µ = 1.32 mm^−^^1^
β = 72.54 (4)°	*T* = 293 K
γ = 69.24 (4)°	Parallelepiped, colourless
*V* = 1373.0 (10) Å^3^	0.20 × 0.12 × 0.04 mm
*Z* = 2	

### Data collection

**Table:**

Oxford Diffraction Xcalibur diffractometer with Ruby CCD	5207 independent reflections
Radiation source: Enhance (Cu) X-ray Source	4503 reflections with *I* > 2σ(*I*)
graphite	*R*_int_ = 0.032
Detector resolution: 10.4922 pixels mm^-1^	θ_max_ = 70.9°, θ_min_ = 3.4°
ο and φ scans	*h* = −12→12
Absorption correction: analytical (*CrysAlis RED*; Oxford Diffraction, 2006)	*k* = −13→13
*T*_min_ = 0.714, *T*_max_ = 0.885	*l* = −16→16
20877 measured reflections	

### Refinement

**Table:**

Refinement on *F*^2^	Primary atom site location: structure-invariant direct methods
Least-squares matrix: full	Secondary atom site location: difference Fourier map
*R*[*F*^2^ > 2σ(*F*^2^)] = 0.041	Hydrogen site location: inferred from neighbouring sites
*wR*(*F*^2^) = 0.128	H-atom parameters not refined
*S* = 1.10	*w* = 1/[σ^2^(*F*_o_^2^) + (0.0892*P*)^2^ + 0.105*P*] where *P* = (*F*_o_^2^ + 2*F*_c_^2^)/3
5207 reflections	(Δ/σ)_max_ = 0.001
352 parameters	Δρ_max_ = 0.33 e Å^−^^3^
0 restraints	Δρ_min_ = −0.24 e Å^−^^3^

### Special details

**Table:**

Experimental. Analytical numeric absorption correction using a multifaceted crystal model based on expressions derived by R.C. Clark & J.S. Reid. (Clark, R. C. & Reid, J. S. (1995). Acta Cryst. A51, 887–897)
Geometry. All e.s.d.'s (except the e.s.d. in the dihedral angle between two l.s. planes) are estimated using the full covariance matrix. The cell e.s.d.'s are taken into account individually in the estimation of e.s.d.'s in distances, angles and torsion angles; correlations between e.s.d.'s in cell parameters are only used when they are defined by crystal symmetry. An approximate (isotropic) treatment of cell e.s.d.'s is used for estimating e.s.d.'s involving l.s. planes.
Refinement. Refinement of *F*^2^ against ALL reflections. The weighted *R*-factor *wR* and goodness of fit *S* are based on *F*^2^, conventional *R*-factors *R* are based on *F*, with *F* set to zero for negative *F*^2^. The threshold expression of *F*^2^ > σ(*F*^2^) is used only for calculating *R*-factors(gt) *etc*. and is not relevant to the choice of reflections for refinement. *R*-factors based on *F*^2^ are statistically about twice as large as those based on *F*, and *R*- factors based on ALL data will be even larger.

### Fractional atomic coordinates and isotropic or equivalent isotropic displacement parameters (Å^2^)

**Table:**

	*x*	*y*	*z*	*U*_iso_*/*U*_eq_	
P1	0.87807 (4)	0.28700 (3)	0.28773 (3)	0.03454 (13)	
O1	0.74905 (14)	0.07868 (13)	0.47544 (10)	0.0626 (4)	
O2	0.56979 (14)	0.26500 (16)	0.47158 (9)	0.0707 (4)	
O3	0.38795 (12)	0.44323 (14)	0.23622 (10)	0.0600 (3)	
O4	0.45565 (13)	0.23001 (13)	0.31754 (11)	0.0608 (3)	
O5	0.52801 (15)	0.67511 (12)	0.19216 (10)	0.0620 (4)	
O6	0.58094 (12)	0.56597 (11)	0.06301 (8)	0.0463 (3)	
O7	0.85225 (13)	0.62633 (11)	0.08444 (10)	0.0549 (3)	
O8	1.01365 (12)	0.49540 (11)	0.17693 (9)	0.0489 (3)	
C1	0.72232 (15)	0.22390 (14)	0.31095 (11)	0.0364 (3)	
C2	0.61807 (15)	0.34781 (15)	0.26462 (11)	0.0383 (3)	
C3	0.67344 (15)	0.44912 (14)	0.20778 (11)	0.0368 (3)	
C4	0.81626 (15)	0.43265 (14)	0.20266 (11)	0.0381 (3)	
C5	0.68130 (17)	0.17803 (17)	0.42808 (12)	0.0458 (4)	
C6	0.5292 (3)	0.2358 (4)	0.58405 (17)	0.1184 (13)	
H6A	0.4466	0.3048	0.6076	0.178*	
H6B	0.6044	0.2319	0.6123	0.178*	
H6C	0.5097	0.1513	0.6065	0.178*	
C7	0.47704 (16)	0.34920 (17)	0.26968 (12)	0.0439 (4)	
C8	0.3211 (2)	0.2186 (3)	0.3245 (2)	0.0829 (7)	
H8A	0.3171	0.1304	0.3599	0.124*	
H8B	0.3063	0.2346	0.2560	0.124*	
H8C	0.2495	0.2833	0.3621	0.124*	
C9	0.58512 (16)	0.57734 (15)	0.15500 (12)	0.0413 (3)	
C10	0.4878 (2)	0.6777 (2)	0.00915 (16)	0.0659 (5)	
H10A	0.4926	0.6597	−0.0564	0.099*	
H10B	0.5155	0.7566	−0.0021	0.099*	
H10C	0.3934	0.6914	0.0500	0.099*	
C11	0.89050 (16)	0.52834 (14)	0.14811 (11)	0.0398 (3)	
C12	1.1076 (2)	0.5725 (2)	0.11739 (17)	0.0631 (5)	
H12A	1.1909	0.5410	0.1442	0.095*	
H12B	1.0622	0.6650	0.1219	0.095*	
H12C	1.1326	0.5632	0.0463	0.095*	
C13	0.76060 (16)	0.11180 (15)	0.24985 (12)	0.0417 (3)	
C14	0.79602 (19)	0.14481 (18)	0.14288 (14)	0.0520 (4)	
H14	0.7991	0.2313	0.1112	0.062*	
C15	0.8269 (2)	0.0515 (2)	0.08212 (17)	0.0666 (5)	
H15	0.8517	0.0751	0.0103	0.080*	
C16	0.8210 (3)	−0.0755 (2)	0.1277 (2)	0.0758 (6)	
H16	0.8411	−0.1382	0.0870	0.091*	
C17	0.7855 (3)	−0.1097 (2)	0.2332 (2)	0.0795 (7)	
H17	0.7814	−0.1961	0.2641	0.095*	
C18	0.7554 (2)	−0.01671 (18)	0.29509 (17)	0.0617 (5)	
H18	0.7319	−0.0413	0.3669	0.074*	
C19	1.03797 (15)	0.16713 (15)	0.24022 (12)	0.0386 (3)	
C20	1.07591 (19)	0.03839 (16)	0.29863 (14)	0.0514 (4)	
H20	1.0197	0.0152	0.3630	0.062*	
C21	1.1982 (2)	−0.05435 (18)	0.25964 (19)	0.0651 (5)	
H21	1.2241	−0.1404	0.2981	0.078*	
C22	1.2813 (2)	−0.0205 (2)	0.16489 (19)	0.0674 (5)	
H22	1.3643	−0.0830	0.1401	0.081*	
C23	1.24248 (19)	0.1049 (2)	0.10654 (15)	0.0586 (5)	
H23	1.2988	0.1268	0.0418	0.070*	
C24	1.11999 (17)	0.19945 (16)	0.14305 (12)	0.0452 (4)	
H24	1.0931	0.2840	0.1026	0.054*	
C25	0.88459 (16)	0.32367 (15)	0.40546 (12)	0.0422 (3)	
C26	0.97926 (18)	0.24694 (18)	0.46578 (13)	0.0492 (4)	
H26	1.0461	0.1693	0.4469	0.059*	
C27	0.9748 (2)	0.2854 (2)	0.55454 (15)	0.0661 (5)	
H27	1.0402	0.2343	0.5944	0.079*	
C28	0.8755 (3)	0.3978 (3)	0.58435 (17)	0.0789 (6)	
H28	0.8729	0.4227	0.6444	0.095*	
C29	0.7807 (3)	0.4726 (3)	0.5259 (2)	0.0965 (9)	
H29	0.7122	0.5484	0.5467	0.116*	
C30	0.7848 (3)	0.4378 (2)	0.43626 (18)	0.0789 (7)	
H30	0.7203	0.4910	0.3961	0.095*	

### Atomic displacement parameters (Å^2^)

**Table:**

	*U*^11^	*U*^22^	*U*^33^	*U*^12^	*U*^13^	*U*^23^
P1	0.0331 (2)	0.0310 (2)	0.0361 (2)	−0.00411 (15)	−0.01189 (14)	−0.00373 (14)
O1	0.0660 (8)	0.0580 (8)	0.0472 (7)	−0.0097 (7)	−0.0169 (6)	0.0087 (6)
O2	0.0576 (8)	0.0871 (10)	0.0400 (6)	0.0074 (7)	−0.0074 (6)	−0.0113 (6)
O3	0.0386 (6)	0.0697 (8)	0.0637 (8)	−0.0056 (6)	−0.0192 (5)	−0.0055 (6)
O4	0.0423 (6)	0.0612 (8)	0.0795 (9)	−0.0201 (6)	−0.0157 (6)	−0.0060 (6)
O5	0.0722 (8)	0.0435 (6)	0.0633 (7)	0.0130 (6)	−0.0303 (6)	−0.0223 (6)
O6	0.0543 (6)	0.0410 (6)	0.0410 (6)	−0.0028 (5)	−0.0215 (5)	−0.0068 (4)
O7	0.0622 (7)	0.0400 (6)	0.0618 (7)	−0.0175 (6)	−0.0285 (6)	0.0100 (5)
O8	0.0456 (6)	0.0440 (6)	0.0573 (7)	−0.0155 (5)	−0.0196 (5)	0.0015 (5)
C1	0.0332 (7)	0.0361 (7)	0.0372 (7)	−0.0083 (6)	−0.0099 (5)	−0.0031 (6)
C2	0.0346 (7)	0.0391 (7)	0.0380 (7)	−0.0039 (6)	−0.0117 (6)	−0.0077 (6)
C3	0.0379 (7)	0.0347 (7)	0.0356 (7)	−0.0014 (6)	−0.0139 (6)	−0.0093 (6)
C4	0.0395 (7)	0.0309 (7)	0.0404 (7)	−0.0051 (6)	−0.0144 (6)	−0.0026 (5)
C5	0.0428 (8)	0.0503 (9)	0.0406 (8)	−0.0146 (7)	−0.0097 (6)	−0.0017 (7)
C6	0.0901 (19)	0.162 (3)	0.0423 (11)	0.0193 (19)	−0.0018 (11)	−0.0134 (15)
C7	0.0366 (7)	0.0540 (9)	0.0395 (7)	−0.0072 (7)	−0.0096 (6)	−0.0133 (7)
C8	0.0564 (12)	0.0940 (17)	0.1114 (19)	−0.0385 (12)	−0.0180 (12)	−0.0192 (15)
C9	0.0412 (8)	0.0369 (7)	0.0422 (8)	−0.0004 (6)	−0.0156 (6)	−0.0099 (6)
C10	0.0837 (14)	0.0527 (10)	0.0630 (11)	−0.0071 (10)	−0.0465 (11)	0.0017 (9)
C11	0.0455 (8)	0.0315 (7)	0.0417 (7)	−0.0076 (6)	−0.0149 (6)	−0.0054 (6)
C12	0.0531 (10)	0.0639 (11)	0.0758 (13)	−0.0278 (9)	−0.0145 (9)	−0.0047 (10)
C13	0.0370 (7)	0.0365 (7)	0.0516 (8)	−0.0076 (6)	−0.0147 (6)	−0.0077 (6)
C14	0.0590 (10)	0.0472 (9)	0.0503 (9)	−0.0108 (8)	−0.0158 (8)	−0.0129 (7)
C15	0.0774 (13)	0.0631 (12)	0.0621 (11)	−0.0089 (10)	−0.0212 (10)	−0.0260 (9)
C16	0.0861 (15)	0.0580 (12)	0.0943 (17)	−0.0055 (11)	−0.0352 (13)	−0.0363 (12)
C17	0.0990 (17)	0.0417 (10)	0.1046 (19)	−0.0212 (11)	−0.0300 (14)	−0.0160 (11)
C18	0.0737 (12)	0.0437 (9)	0.0665 (11)	−0.0186 (9)	−0.0187 (10)	−0.0042 (8)
C19	0.0345 (7)	0.0354 (7)	0.0441 (7)	−0.0036 (6)	−0.0146 (6)	−0.0075 (6)
C20	0.0491 (9)	0.0397 (8)	0.0572 (10)	−0.0055 (7)	−0.0166 (8)	−0.0014 (7)
C21	0.0596 (11)	0.0380 (9)	0.0876 (14)	0.0051 (8)	−0.0289 (11)	−0.0087 (9)
C22	0.0480 (10)	0.0590 (11)	0.0890 (15)	0.0070 (9)	−0.0155 (10)	−0.0343 (11)
C23	0.0475 (9)	0.0654 (11)	0.0585 (10)	−0.0068 (8)	−0.0037 (8)	−0.0272 (9)
C24	0.0459 (8)	0.0445 (8)	0.0433 (8)	−0.0083 (7)	−0.0120 (7)	−0.0099 (7)
C25	0.0444 (8)	0.0421 (8)	0.0414 (7)	−0.0109 (7)	−0.0132 (6)	−0.0085 (6)
C26	0.0490 (9)	0.0516 (9)	0.0455 (8)	−0.0133 (8)	−0.0167 (7)	−0.0026 (7)
C27	0.0751 (13)	0.0788 (14)	0.0506 (10)	−0.0234 (11)	−0.0307 (9)	−0.0029 (9)
C28	0.1066 (18)	0.0837 (15)	0.0560 (11)	−0.0224 (14)	−0.0288 (12)	−0.0244 (11)
C29	0.116 (2)	0.0873 (17)	0.0825 (16)	0.0170 (16)	−0.0434 (15)	−0.0498 (14)
C30	0.0877 (15)	0.0693 (13)	0.0761 (14)	0.0194 (12)	−0.0430 (12)	−0.0377 (11)

### Geometric parameters (Å, °)

**Table:**

P1—C4	1.7342 (17)	C12—H12C	0.9600
P1—C19	1.7872 (19)	C13—C18	1.379 (2)
P1—C25	1.8001 (16)	C13—C14	1.383 (2)
P1—C1	1.8921 (17)	C14—C15	1.384 (3)
O1—C5	1.201 (2)	C14—H14	0.9300
O2—C5	1.314 (2)	C15—C16	1.367 (3)
O2—C6	1.453 (3)	C15—H15	0.9300
O3—C7	1.204 (2)	C16—C17	1.365 (4)
O4—C7	1.347 (2)	C16—H16	0.9300
O4—C8	1.426 (2)	C17—C18	1.395 (3)
O5—C9	1.192 (2)	C17—H17	0.9300
O6—C9	1.3220 (19)	C18—H18	0.9300
O6—C10	1.440 (2)	C19—C24	1.384 (2)
O7—C11	1.2078 (19)	C19—C20	1.395 (2)
O8—C11	1.357 (2)	C20—C21	1.384 (3)
O8—C12	1.436 (2)	C20—H20	0.9300
C1—C2	1.521 (2)	C21—C22	1.369 (3)
C1—C5	1.526 (2)	C21—H21	0.9300
C1—C13	1.543 (2)	C22—C23	1.369 (3)
C2—C3	1.367 (2)	C22—H22	0.9300
C2—C7	1.448 (2)	C23—C24	1.385 (3)
C3—C4	1.420 (2)	C23—H23	0.9300
C3—C9	1.503 (2)	C24—H24	0.9300
C4—C11	1.429 (2)	C25—C26	1.375 (2)
C6—H6A	0.9600	C25—C30	1.386 (3)
C6—H6B	0.9600	C26—C27	1.382 (3)
C6—H6C	0.9600	C26—H26	0.9300
C8—H8A	0.9600	C27—C28	1.366 (3)
C8—H8B	0.9600	C27—H27	0.9300
C8—H8C	0.9600	C28—C29	1.355 (4)
C10—H10A	0.9600	C28—H28	0.9300
C10—H10B	0.9600	C29—C30	1.374 (3)
C10—H10C	0.9600	C29—H29	0.9300
C12—H12A	0.9600	C30—H30	0.9300
C12—H12B	0.9600		
			
C4—P1—C19	118.31 (8)	O8—C12—H12C	109.5
C4—P1—C25	111.01 (8)	H12A—C12—H12C	109.5
C19—P1—C25	110.45 (8)	H12B—C12—H12C	109.5
C4—P1—C1	95.25 (8)	C18—C13—C14	118.39 (16)
C19—P1—C1	110.67 (8)	C18—C13—C1	124.08 (16)
C25—P1—C1	110.13 (8)	C14—C13—C1	117.46 (14)
C5—O2—C6	116.29 (17)	C13—C14—C15	121.17 (18)
C7—O4—C8	116.36 (17)	C13—C14—H14	119.4
C9—O6—C10	115.99 (13)	C15—C14—H14	119.4
C11—O8—C12	116.43 (14)	C16—C15—C14	120.0 (2)
C2—C1—C5	115.65 (13)	C16—C15—H15	120.0
C2—C1—C13	109.52 (12)	C14—C15—H15	120.0
C5—C1—C13	113.31 (13)	C17—C16—C15	119.67 (19)
C2—C1—P1	101.49 (10)	C17—C16—H16	120.2
C5—C1—P1	105.46 (10)	C15—C16—H16	120.2
C13—C1—P1	110.62 (10)	C16—C17—C18	120.8 (2)
C3—C2—C7	123.29 (14)	C16—C17—H17	119.6
C3—C2—C1	114.70 (13)	C18—C17—H17	119.6
C7—C2—C1	121.52 (14)	C13—C18—C17	120.0 (2)
C2—C3—C4	118.33 (14)	C13—C18—H18	120.0
C2—C3—C9	121.37 (14)	C17—C18—H18	120.0
C4—C3—C9	120.26 (14)	C24—C19—C20	119.92 (15)
C3—C4—C11	125.85 (14)	C24—C19—P1	120.09 (12)
C3—C4—P1	108.19 (12)	C20—C19—P1	119.89 (13)
C11—C4—P1	125.44 (12)	C21—C20—C19	119.24 (18)
O1—C5—O2	124.05 (16)	C21—C20—H20	120.4
O1—C5—C1	123.65 (15)	C19—C20—H20	120.4
O2—C5—C1	112.10 (14)	C22—C21—C20	120.56 (18)
O2—C6—H6A	109.5	C22—C21—H21	119.7
O2—C6—H6B	109.5	C20—C21—H21	119.7
H6A—C6—H6B	109.5	C23—C22—C21	120.22 (17)
O2—C6—H6C	109.5	C23—C22—H22	119.9
H6A—C6—H6C	109.5	C21—C22—H22	119.9
H6B—C6—H6C	109.5	C22—C23—C24	120.53 (18)
O3—C7—O4	122.88 (15)	C22—C23—H23	119.7
O3—C7—C2	125.66 (17)	C24—C23—H23	119.7
O4—C7—C2	111.46 (14)	C23—C24—C19	119.47 (16)
O4—C8—H8A	109.5	C23—C24—H24	120.3
O4—C8—H8B	109.5	C19—C24—H24	120.3
H8A—C8—H8B	109.5	C26—C25—C30	118.85 (16)
O4—C8—H8C	109.5	C26—C25—P1	124.91 (13)
H8A—C8—H8C	109.5	C30—C25—P1	116.24 (13)
H8B—C8—H8C	109.5	C25—C26—C27	119.85 (18)
O5—C9—O6	125.55 (14)	C25—C26—H26	120.1
O5—C9—C3	123.73 (14)	C27—C26—H26	120.1
O6—C9—C3	110.72 (12)	C28—C27—C26	120.73 (19)
O6—C10—H10A	109.5	C28—C27—H27	119.6
O6—C10—H10B	109.5	C26—C27—H27	119.6
H10A—C10—H10B	109.5	C29—C28—C27	119.59 (19)
O6—C10—H10C	109.5	C29—C28—H28	120.2
H10A—C10—H10C	109.5	C27—C28—H28	120.2
H10B—C10—H10C	109.5	C28—C29—C30	120.7 (2)
O7—C11—O8	122.87 (14)	C28—C29—H29	119.6
O7—C11—C4	126.20 (15)	C30—C29—H29	119.6
O8—C11—C4	110.93 (13)	C29—C30—C25	120.2 (2)
O8—C12—H12A	109.5	C29—C30—H30	119.9
O8—C12—H12B	109.5	C25—C30—H30	119.9
H12A—C12—H12B	109.5		
			
C4—P1—C1—C2	−12.94 (10)	C12—O8—C11—O7	−8.6 (2)
C19—P1—C1—C2	−135.88 (10)	C12—O8—C11—C4	171.39 (14)
C25—P1—C1—C2	101.72 (11)	C3—C4—C11—O7	−13.6 (3)
C4—P1—C1—C5	−133.91 (11)	P1—C4—C11—O7	175.67 (13)
C19—P1—C1—C5	103.16 (11)	C3—C4—C11—O8	166.45 (13)
C25—P1—C1—C5	−19.25 (12)	P1—C4—C11—O8	−4.29 (19)
C4—P1—C1—C13	103.22 (11)	C2—C1—C13—C18	−129.20 (17)
C19—P1—C1—C13	−19.72 (12)	C5—C1—C13—C18	1.6 (2)
C25—P1—C1—C13	−142.12 (11)	P1—C1—C13—C18	119.74 (16)
C5—C1—C2—C3	124.77 (14)	C2—C1—C13—C14	47.76 (18)
C13—C1—C2—C3	−105.73 (14)	C5—C1—C13—C14	178.51 (14)
P1—C1—C2—C3	11.24 (14)	P1—C1—C13—C14	−63.30 (17)
C5—C1—C2—C7	−63.05 (18)	C18—C13—C14—C15	−0.6 (3)
C13—C1—C2—C7	66.44 (17)	C1—C13—C14—C15	−177.72 (17)
P1—C1—C2—C7	−176.59 (11)	C13—C14—C15—C16	0.8 (3)
C7—C2—C3—C4	−175.57 (13)	C14—C15—C16—C17	−0.5 (4)
C1—C2—C3—C4	−3.56 (19)	C15—C16—C17—C18	−0.1 (4)
C7—C2—C3—C9	6.7 (2)	C14—C13—C18—C17	0.0 (3)
C1—C2—C3—C9	178.76 (12)	C1—C13—C18—C17	176.93 (18)
C2—C3—C4—C11	−179.26 (14)	C16—C17—C18—C13	0.4 (4)
C9—C3—C4—C11	−1.5 (2)	C4—P1—C19—C24	9.56 (15)
C2—C3—C4—P1	−7.20 (16)	C25—P1—C19—C24	−119.90 (13)
C9—C3—C4—P1	170.51 (10)	C1—P1—C19—C24	117.88 (13)
C19—P1—C4—C3	128.76 (11)	C4—P1—C19—C20	−166.72 (12)
C25—P1—C4—C3	−102.04 (12)	C25—P1—C19—C20	63.82 (15)
C1—P1—C4—C3	11.88 (11)	C1—P1—C19—C20	−58.40 (15)
C19—P1—C4—C11	−59.14 (15)	C24—C19—C20—C21	2.1 (3)
C25—P1—C4—C11	70.06 (15)	P1—C19—C20—C21	178.33 (14)
C1—P1—C4—C11	−176.01 (13)	C19—C20—C21—C22	0.0 (3)
C6—O2—C5—O1	0.0 (3)	C20—C21—C22—C23	−1.5 (3)
C6—O2—C5—C1	−175.0 (2)	C21—C22—C23—C24	0.9 (3)
C2—C1—C5—O1	176.41 (15)	C22—C23—C24—C19	1.2 (3)
C13—C1—C5—O1	48.8 (2)	C20—C19—C24—C23	−2.7 (2)
P1—C1—C5—O1	−72.36 (19)	P1—C19—C24—C23	−178.96 (13)
C2—C1—C5—O2	−8.5 (2)	C4—P1—C25—C26	−149.65 (14)
C13—C1—C5—O2	−136.16 (15)	C19—P1—C25—C26	−16.38 (17)
P1—C1—C5—O2	102.70 (15)	C1—P1—C25—C26	106.15 (16)
C8—O4—C7—O3	1.7 (3)	C4—P1—C25—C30	30.89 (19)
C8—O4—C7—C2	−178.33 (17)	C19—P1—C25—C30	164.16 (17)
C3—C2—C7—O3	−9.5 (2)	C1—P1—C25—C30	−73.31 (19)
C1—C2—C7—O3	179.04 (15)	C30—C25—C26—C27	−0.9 (3)
C3—C2—C7—O4	170.58 (14)	P1—C25—C26—C27	179.64 (15)
C1—C2—C7—O4	−0.9 (2)	C25—C26—C27—C28	1.3 (3)
C10—O6—C9—O5	−6.5 (3)	C26—C27—C28—C29	−0.3 (4)
C10—O6—C9—C3	173.85 (15)	C27—C28—C29—C30	−0.9 (5)
C2—C3—C9—O5	94.9 (2)	C28—C29—C30—C25	1.3 (5)
C4—C3—C9—O5	−82.8 (2)	C26—C25—C30—C29	−0.4 (4)
C2—C3—C9—O6	−85.44 (17)	P1—C25—C30—C29	179.1 (2)
C4—C3—C9—O6	96.92 (17)		
